# An Integrated Machine-Learning and Reverse Network-Pharmacology Pipeline Reveals *JUN*/C3 Candidate Biomarkers and an Anti-Fibrotic Mechanism of Resveratrol via MAPK/JNK Signaling in Chronic Kidney Disease

**DOI:** 10.3390/ijms27104252

**Published:** 2026-05-10

**Authors:** Yuan Cai, Xiaolong Feng, Xinru Tao, Penghui Li, Jiaqin Liu, Ping’an Liu, Mengxiong Xiao

**Affiliations:** 1 Hunan Academy of Chinese Medicine, Changsha 410013, China; tcmyuanyuan@163.com (Y.C.);; 2 Experimental Research Center, China Academy of Chinese Medical Sciences, Beijing 100700, China

**Keywords:** chronic kidney disease, *JUN*, C3, immune inflammation, resveratrol, MAPK/JNK

## Abstract

Chronic kidney disease (CKD) lacks highly specific early diagnostic biomarkers and safe, effective therapeutic options. To address this, we integrated multi-cohort transcriptomics, bioinformatics, and machine learning with reverse network pharmacology, molecular docking, molecular dynamics simulations, and in vivo experiments to identify candidate biomarkers associated with CKD and candidate therapeutic compounds for CKD. Our analyses of the GSE175759 training set and external validation datasets (GSE37171 and GSE66494) using differential expression and WGCNA indicated that CKD is characterized by immune-inflammatory activation and suppressed energy metabolism. Integration of PPI analysis with three machine learning algorithms identified *JUN* and C3 as candidate diagnostic genes. *JUN* was downregulated, whereas C3 was upregulated in CKD, both showing promising discriminatory performance in the analyzed datasets. Reverse screening identified Resveratrol and Triptolide as candidate active compounds, and molecular docking together with 100 ns molecular dynamics simulations supported stable binding to *JUN*/C3 complexes. Given its superior safety profile, Resveratrol was selected for experimental validation. In an adenine-induced CKD rat model, Resveratrol improved renal function, reduced proteinuria, alleviated renal injury and fibrosis, and was associated with reduced activation of MAPK/JNK-c-Jun signaling. In conclusion, this study identifies *JUN* and C3 as candidate biomarkers associated with CKD and suggests Resveratrol as a promising preclinical intervention candidate targeting the MAPK/JNK-c-Jun axis, providing preliminary computational and preclinical evidence for biomarker discovery and natural medicine-based intervention in CKD.

## 1. Introduction

Chronic kidney disease (CKD) has become a global public health challenge. Data from the Global Burden of Disease Study 2023 indicate that CKD currently ranks as the ninth leading cause of death worldwide and affects approximately 13.4% of adults [1]. Its incidence is continuing to rise, largely in parallel with the increasing prevalence of risk factors such as obesity, hypertension, and diabetes [2]. CKD not only imposes an escalating economic burden on health-care systems worldwide but also substantially compromises patients’ quality of life and survival. Notably, patients with CKD have a 10–20-fold higher risk of cardiovascular mortality than the general population [3], and even mild reductions in kidney function are closely associated with increased all-cause mortality [4]. The pathophysiology of CKD is highly complex, involving interactive regulatory networks across multiple genes and pathways. Emerging evidence suggests that fibrotic mechanisms are central drivers of CKD progression [5]. In parallel, dysregulation of the immune–inflammatory microenvironment is a pivotal driver of CKD progression [6]. Specifically, aberrant complement activation and the infiltration of pro-inflammatory cells, such as M1 macrophages, collectively promote renal inflammation and tissue remodeling [7,8,9]. In addition, excessive activation of the renin–angiotensin–aldosterone system (RAAS) is a key contributor to the mechanisms underlying cardiorenal comorbidity [10].

Despite advances in understanding its pathogenesis, early diagnosis of CKD remains challenging [11]. Current clinical guidelines primarily recommend estimated glomerular filtration rate (eGFR) and the urinary albumin-to-creatinine ratio (ACR) for assessment [11,12]; however, these conventional markers are readily influenced by factors such as infection and physical activity and often lack sensitivity in the early stages of disease [12]. Emerging biomarkers like neutrophil gelatinase-associated lipocalin (NGAL) and kidney injury molecule-1 (KIM-1) have shown promise [11,13]. However, their limited accessibility and the ongoing lack of highly specific markers for early diagnosis remain significant challenges. Identifying biomarkers that can detect subclinical kidney injury and enable intervention before substantial declines in renal function is critical for improving patient outcomes.

In recent years, with rapid advances in multi-omics technologies and artificial intelligence (AI), bioinformatics and machine-learning algorithms have become powerful tools for identifying disease biomarkers [14]. Integrative analyses of GEO datasets enable systematic identification of key genes with diagnostic or prognostic value. By performing dimensionality reduction and improving model generalizability, machine-learning approaches facilitate biomarker discovery and help overcome the limitations of conventional single-gene analyses [15].

**Figure 1:**
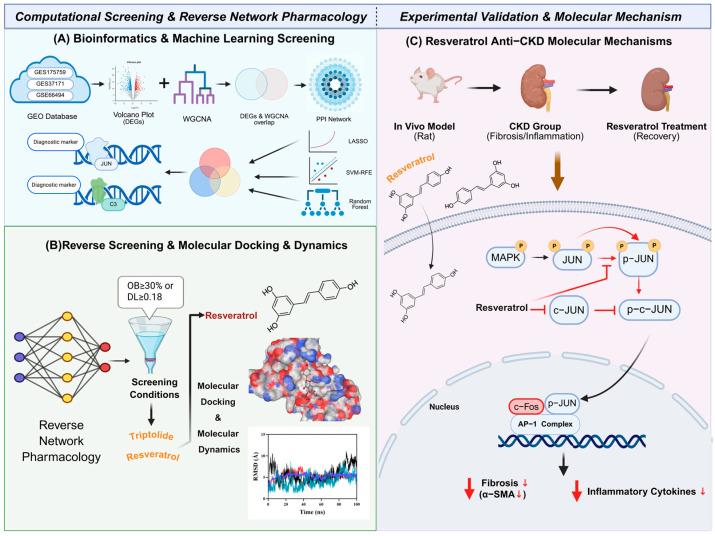
The workflow of the research. Arrows indicate biological activation, signal transduction, or molecular translocation, while downward red arrows indicate a reduction in pathological indicators. Red “T”-shaped lines denote pharmacological inhibition.

To address these challenges, we integrated a multidimensional framework encompassing bioinformatics, machine learning, reverse network pharmacology, molecular simulations, and animal experiments. First, using the GSE175759 dataset as the training set, we identified differentially expressed genes (DEGs) between CKD and healthy controls, constructed a weighted gene co-expression network (WGCNA) and a protein–protein interaction (PPI) network, and applied three machine-learning algorithms to pinpoint the core promising diagnostic candidates *JUN* and C3. Subsequently, two independent datasets (GSE37171 and GSE66494) were utilized as external validation sets to verify their expression patterns and discriminatory potential. Next, a reverse network pharmacology strategy was used to prioritize the bioactive natural compounds resveratrol and triptolide targeting these core genes, and molecular docking together with molecular dynamics simulations supported the stability of their interactions with the target proteins. Finally, using an adenine-induced fibrotic CKD rat model, we verified the expression patterns of these core genes and evaluated the therapeutic effects of Resveratrol. We further investigated whether the MAPK/JNK–c-Jun signaling axis mediates Resveratrol’s anti-fibrotic activity. This study provides preliminary evidence for CKD biomarker discovery and establishes a foundation for future clinical translation. The study workflow is summarized in Figure 1.

## 2. Results

### 2.1. Identification of DEGs in CKD and Construction of WGCNA

**Figure 2:**
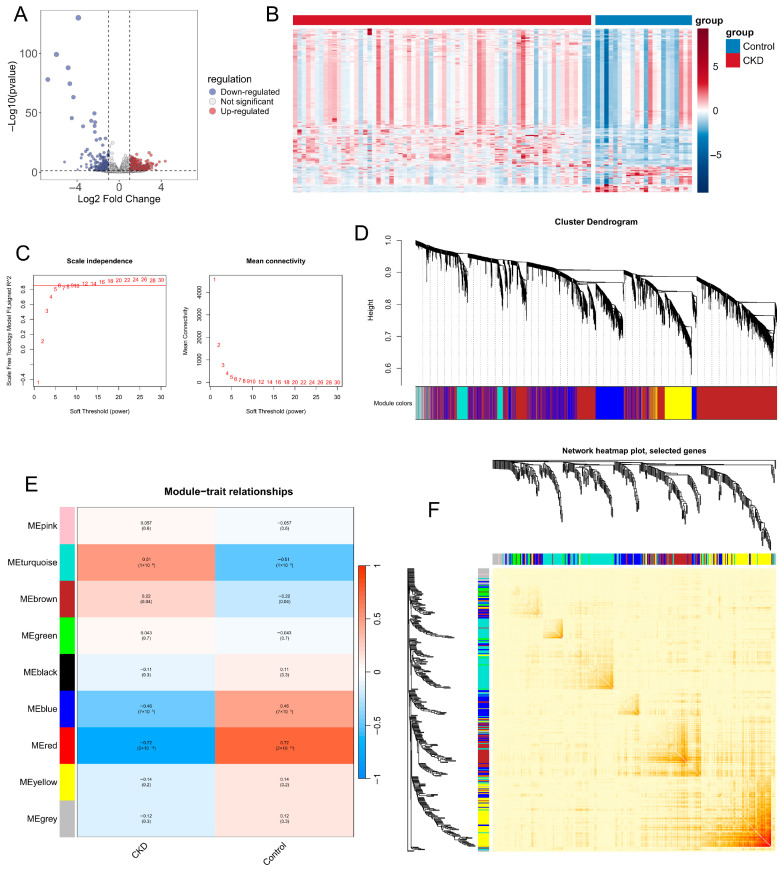
Differential expression analysis and construction of a weighted gene co-expression network in the GSE175759 cohort. (**A**) Volcano plot of DEGs between CKD and healthy controls (FC > 1.5 and FDR-adjusted *p* < 0.05); red indicates upregulated genes, blue indicates downregulated genes, and gray indicates non-significant genes. (**B**) Heatmap of DEGs showing distinct expression patterns between CKD and control samples; the top annotation bar denotes sample groups, and colors represent normalized expression levels. (**C**) Determination of the soft-thresholding power (β) for WGCNA based on scale-free topology fitting (**left**) and mean connectivity (**right**). The red numbers inside the plots represent the evaluated candidate soft-thresholding powers. β = 6 was selected for subsequent network construction. (**D**) Hierarchical clustering dendrogram of genes. Each color in the band below represents a distinct co-expression module. (**E**) Module–trait relationship heatmap displaying correlations between module eigengenes and clinical traits (CKD vs. control); numbers in each cell represent Pearson correlation coefficients with corresponding *p*-values in parentheses. (**F**) TOM heatmap of selected genes. The yellow-to-red gradient indicates low-to-high connectivity, with module colors on the axes.

To identify potential biomarkers associated with CKD progression, we first analyzed gene expression profiles from the GSE175759 dataset. Differential expression analysis was performed using the “limma” package in R, with DEGs screened using thresholds of FC > 1.5 and an adjusted *p*-value < 0.05. The volcano plot showed the distribution of DEGs, with red and blue points indicating significantly upregulated and downregulated genes, respectively (Figure 2A). The heatmap further revealed distinct DEG expression patterns between CKD patients and control groups (Figure 2B). To identify CKD-related co-expression modules, we constructed a weighted gene co-expression network using WGCNA. The optimal soft-thresholding power was set to β = 6, at which the scale-free topology fit index approached 0.9 and mean connectivity became stable, indicating that the network met the scale-free topology criterion (Figure 2C). Genes with similar expression patterns were then clustered into distinct modules using hierarchical clustering and the dynamic tree cut algorithm (Figure 2D). Module-trait correlation analysis showed that the turquoise module was positively correlated with CKD (r = 0.51, *p* = 1 × 10^−6^), whereas the red module showed the strongest negative correlation (r = −0.72, *p* = 2 ×10^−15^) (Figure 2E). In both modules, Gene Significance for CKD was strongly correlated with Module Membership, supporting their relevance to CKD progression (Figure S1A,B). Finally, the topological overlap matrix heatmap showed high intra-module connectivity and clear module separation, confirming the robustness of the module classification (Figure 2F).

### 2.2. Biological Functions of Core Intersection Genes and Characteristics of the Immune

**Figure 3:**
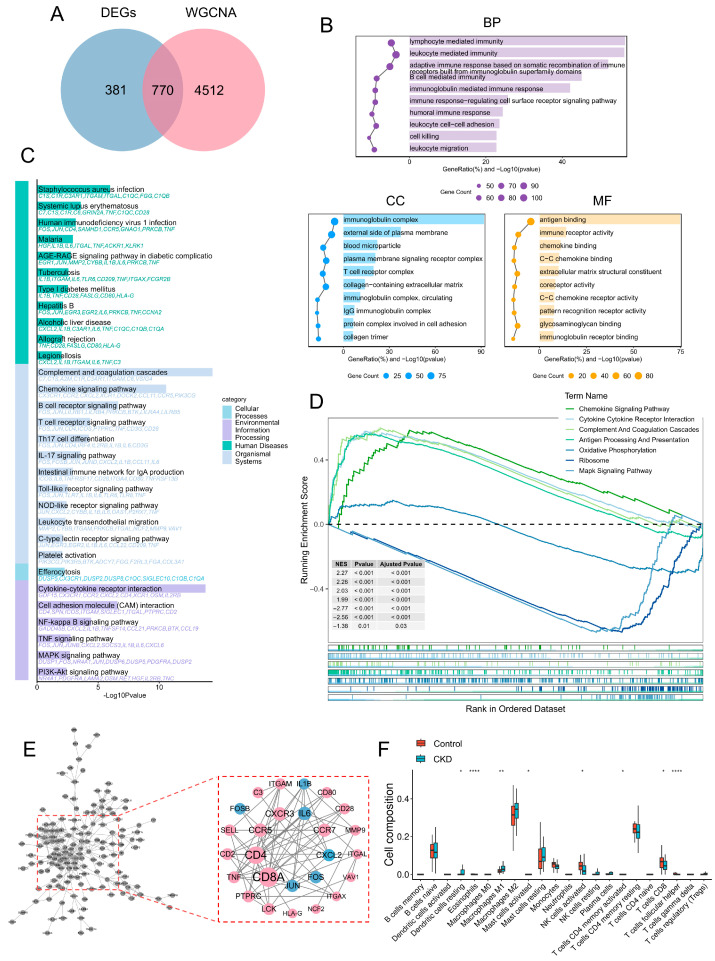
Functional enrichment, protein interaction network, and immune infiltration characteristics of CKD-associated intersecting genes. (**A**) Venn diagram showing the overlap between DEGs and WGCNA-derived module genes, yielding 770 candidate genes. (**B**) GO enrichment of the 770 genes across BP, CC, and MF. (**C**) KEGG pathway enrichment analysis of the intersecting genes. (**D**) GSEA highlighting activated immune–inflammatory pathways and suppressed metabolic/protein synthesis pathways in CKD. (**E**) PPI network of intersecting genes constructed using STRING and visualized in Cytoscape; hub genes were identified by topological analysis (*n* = 26). (**F**) Immune cell infiltration estimated by CIBERSORT; boxplots compare immune cell fractions between CKD and controls. Data are presented as mean ± SD. * *p* < 0.05, ** *p* < 0.01, **** *p* < 0.001.

To refine the key genes associated with CKD progression, DEGs were intersected with WGCNA-derived key module genes, yielding 770 candidate genes (Figure 3A). GO enrichment analysis showed that these genes were mainly involved in immune-related biological processes, including lymphocyte-mediated immunity, leukocyte-mediated immunity, and adaptive immune responses (Figure 3B). In the Cellular Components (CC) category, they were primarily enriched in immunoglobulin complexes and the external side of the plasma membrane, while Molecular Functions (MF) analysis indicated significant enrichment in antigen binding, chemokine binding, and immune receptor activity. KEGG pathway analysis further showed that these genes were enriched in immune- and inflammation-related pathways, including Staphylococcus aureus infection, systemic lupus erythematosus, chemokine signaling, cytokine-cytokine receptor interaction, NF-κB signaling, and TNF signaling pathways (Figure 3C). To further characterize pathway alterations in CKD, GSEA revealed significant activation of the chemokine signaling pathway, cytokine-cytokine receptor interaction, and complement and coagulation cascades in the CKD group (NES > 1.5, *p* < 0.05) (Figure 3D). In contrast, oxidative phosphorylation and ribosome-related pathways were negatively enriched (NES < −2.0, *p* < 0.05), suggesting that CKD progression may involve both immune-inflammatory activation and impaired mitochondrial metabolism. A PPI network was then constructed using the STRING database and Cytoscape 3.10.1 to explore interactions among these candidate genes (Figure 3E). Topological analysis identified 26 core interacting genes, including CD4, CD8A, IL6, IL1B, CXCR3, CCR5, ITGAM, PTPRC, and *JUN*, which occupied central positions in the network and may contribute to immune-inflammatory dysregulation in CKD. Given the prominent immune-related enrichment, CIBERSORT was used to evaluate immune cell infiltration in CKD samples (Figure 3F). Compared with the Control group, the CKD group showed significantly increased proportions of pro-inflammatory immune cells, including M1 macrophages, activated CD4^+^ memory T cells, and activated mast cells, whereas CD8^+^ T cells and activated NK cells were significantly reduced (*p* < 0.05). Overall, the immune landscape was characterized by enhanced pro-inflammatory cell infiltration and reduced cytotoxic immune cell populations, which was consistent with the activation of inflammatory pathways and the identification of immune-related hub genes. These findings suggest that aberrant immune cell infiltration may be an important pathological feature of CKD progression.

### 2.3. Screening and Evaluation of Promising Diagnostic Candidates for CKD Based on Machine Learning Algorithms

**Figure 4:**
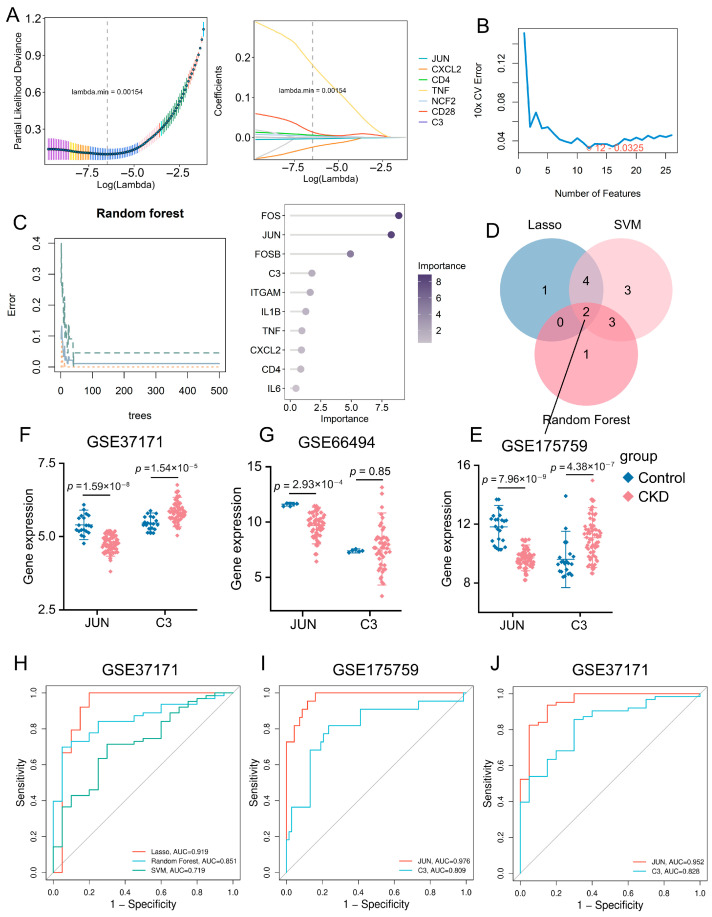
Machine learning–based identification and evaluation of candidate biomarkers for CKD. (**A**) LASSO regression with 10-fold cross-validation to determine the optimal penalty (λ_min_) and select 7 feature genes. (**B**) SVM-RFE analysis identifying the optimal 12-gene subset with the lowest classification error. (**C**) Random forest model performance. Left: Out-of-bag (OOB) error rate as a function of the number of trees. The line represents the overall OOB error. Right: Variable importance ranking of the top 10 genes. (**D**) Venn diagram showing the intersection of genes screened by LASSO, SVM-RFE, and Random Forest, yielding two hub biomarkers (*JUN* and C3). (**E**–**G**) Expression of *JUN* and C3 in the training cohort (GSE175759) and validation cohorts (GSE37171, GSE66494). (**H**) ROC curves comparing model performance in GSE37171. (**I**,**J**) ROC curves of single genes in GSE175759.

To further screen potential diagnostic biomarkers for CKD, three machine-learning algorithms, including LASSO regression, SVM-RFE, and Random Forest, were applied to the 26 PPI-derived core genes. LASSO, SVM-RFE, and Random Forest identified 7, 12, and 10 candidate genes, respectively (Figure 4A–C). Following the intersection of genes obtained from the three algorithms, two promising diagnostic candidates were ultimately identified: *JUN* and C3 (Figure 4D); notably, *JUN* was significantly downregulated, while C3 was significantly upregulated in the CKD group (Figure 4E). In the external validation set GSE37171, *JUN* remained significantly downregulated, and C3 was significantly upregulated in the CKD group (Figure 4F). In the external validation set GSE66494, the difference in C3 expression between groups was not statistically significant (*p* = 0.85); this may be attributed to the limited sample size of the control group (*n* = 5), which likely resulted in insufficient statistical power. Nevertheless, *JUN* was consistently downregulated in the CKD group (Figure 4G).

To assess the discriminatory capability of the core genes, ROC curves were generated and the corresponding AUC values were calculated. In GSE37171, the LASSO model showed the highest discriminatory performance with an AUC of 0.919, compared with the Random Forest model (AUC = 0.851) and the SVM model (AUC = 0.719) (Figure 4H). Single genes’ ROC analysis further showed that, in the training dataset GSE175759, the AUC values of *JUN* and C3 were 0.976 and 0.809, respectively (Figure 4I). In the external validation dataset GSE37171, the AUC values of *JUN* and C3 were 0.952 and 0.828, respectively (Figure 4J). The confidence intervals, standard errors, *p*-values, sensitivity, specificity, and Youden indices of the AUC values are summarized in Supplementary Table S1. Detailed ROC analysis was not performed on the GSE66494 dataset due to the limited number of normal controls, and this dataset was therefore used only for supplementary expression validation. Overall, *JUN* showed favorable discriminatory ability across the training and validation sets, while C3 also exhibited potential diagnostic value in the analyzed datasets, although its result in GSE66494 should be interpreted cautiously. An exploratory CKD risk prediction nomogram based on *JUN* and C3 was further constructed to visualize their potential contribution to CKD discrimination (Figure S2).

### 2.4. GSEA Enrichment Analysis of Core Genes

To further explore the biological processes associated with the promising diagnostic candidates, single-gene GSEA was performed for *JUN* and C3. For *JUN*, positive enriched gene sets were mainly involved in immune-inflammatory and stress-related signaling pathways, involving the MAPK signaling pathway, Toll-like receptor signaling pathway, graft-versus-host disease, and infection-related pathways. In contrast, *JUN*-associated gene sets were negatively enriched in metabolic and detoxification-related processes, such as retinol metabolism, tryptophan metabolism, propanoate metabolism, and cytochrome P450-mediated drug metabolism (Figure S3A). For C3, positive enriched gene sets were mainly associated with immune activation and cell–cell interactions, including cytokine-cytokine receptor interaction, ECM-receptor interaction, hematopoietic cell lineage, systemic lupus erythematosus, and cell adhesion molecules. Conversely, C3-associated gene sets were negatively enriched in energy metabolism and protein synthesis pathways, including oxidative phosphorylation and ribosome-related pathways, as well as mitochondrial dysfunction-related neurodegenerative disease pathways (Figure S3B). Collectively, single-gene GSEA of *JUN* and C3 further supported the finding that CKD is characterized by enhanced immune-inflammatory activation and impaired mitochondrial energy metabolism/protein synthesis, thereby providing functional evidence for the preceding enrichment analyses and candidate diagnostic biomarker screening.

### 2.5. Reverse Network Pharmacology Screening of Key Targets and Validation via Molecular Docking and Molecular Dynamics

**Figure 5:**
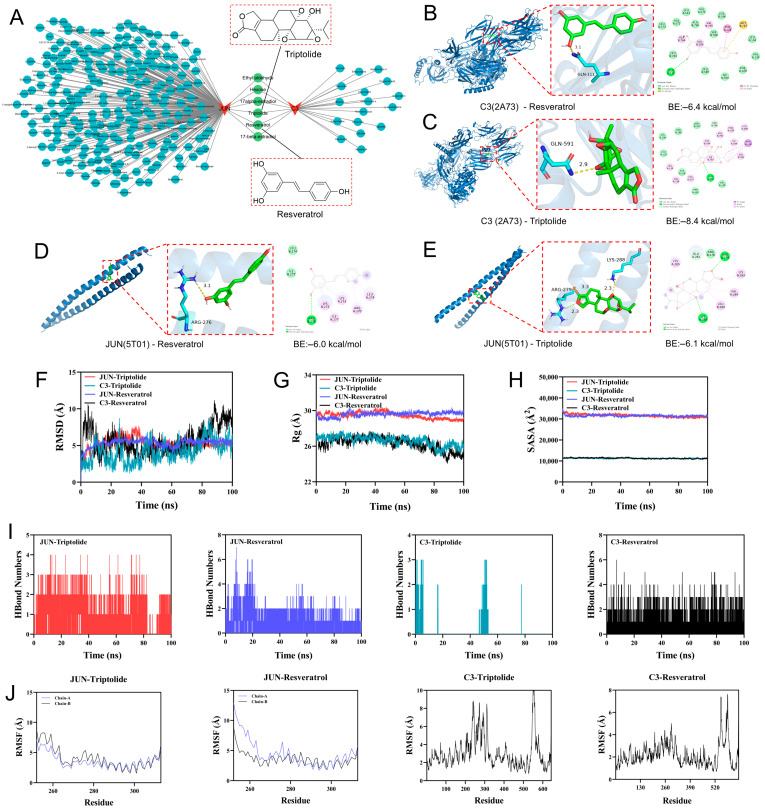
Reverse network pharmacology screening and molecular docking/MD validation of candidate compounds targeting *JUN* and C3. (**A**) “Compound-target” interaction network generated by reverse screening for *JUN* and C3; six compounds were shared by both targets, with Triptolide and Resveratrol highlighted. (**B**–**E**) Representative docking poses and 2D interaction maps of Resveratrol/Triptolide with C3 (PDB: 2A73) and *JUN* (PDB: 5T01); binding energies (kcal/mol) are shown. (**F**–**H**) MD trajectories (100 ns) of the four complexes showing RMSD, Rg, and SASA. (**I**) Time evolution of hydrogen-bond numbers during MD simulations. (**J**) Residue-wise RMSF profiles of the complexes, indicating overall conformational stability upon ligand binding.

To identify potential active compounds targeting the promising diagnostic candidates, we used a reverse network pharmacology strategy to screen compounds associated with *JUN* and C3 and constructed a compound-target interaction network. A total of 167 compounds were predicted to target *JUN*, whereas 23 compounds were predicted to target C3; among them, six compounds were shared by both targets (Figure 5A). Two natural bioactive monomers, Triptolide and Resveratrol, were selected for further analysis. Triptolide is mainly derived from *Tripterygium wilfordii* Hook. f., whereas Resveratrol is commonly found in plants such as *Polygonum cuspidatum* Sieb. et Zucc. Previous studies have reported their renoprotective potential in CKD-related models, supporting their further evaluation as candidate active compounds.

Molecular docking was then performed to assess the binding potential of Triptolide and Resveratrol with *JUN* and C3. The results showed that both compounds could fit into the predicted binding pockets and form hydrogen bonds or hydrophobic interactions with the target proteins. For C3, the binding energy of the C3-Triptolide complex was −8.4 kcal/mol, which was lower than that of the C3-Resveratrol complex (−6.4 kcal/mol), suggesting a stronger predicted affinity of Triptolide for C3 (Figure 5B,C). For *JUN*, the binding energies of *JUN*-Triptolide and *JUN*-Resveratrol were −6.1 and −6.0 kcal/mol, respectively, indicating comparable binding affinities (Figure 5D,E). Binding modes analysis further showed that these ligands interacted with key residues, including GLN-111 and GLN-591 in C3, as well as ARG-276 and LYS-288 in the *JUN*, providing structural support for their potential target interactions (Figure 5B–E).

Based on the docking results, 100 ns molecular dynamics (MD) simulations were further performed to evaluate the dynamic stability of the *JUN*-ligand and C3-ligand complexes. Root Mean Square Deviation (RMSD) analysis showed that the *JUN*-Triptolide complex reached stability after approximately 45 ns, fluctuating around 6 Å, whereas the *JUN*-Resveratrol complex reached equilibrium after 70 ns, stabilizing at approximately 5.5 Å (Figure 5F). The Radius of Gyration (Rg) and Solvent Accessible Surface Area (SASA) showed only minor fluctuations, suggesting that ligand binding did not markedly alter the overall compactness or surface exposure of *JUN* (Figure 5G,H). Hydrogen bond analysis showed that both complexes maintained approximately two hydrogen bonds during most of the simulation period, indicating stable non-covalent interactions (Figure 5I). Root Mean Square Fluctuation (RMSF) analysis also revealed generally low residue flexibility, further supporting the stability of the two *JUN*-ligand complexes (Figure 5J). Collectively, molecular docking suggested that Triptolide and Resveratrol could bind to both *JUN* and C3, while MD simulations further confirmed the dynamic stability of the *JUN*–Triptolide and *JUN*–Resveratrol complexes, providing computational evidence for subsequent experimental validation.

### 2.6. Establishment of the Adenine-Induced CKD Model and the Renoprotective Effects of Resveratrol

**Figure 6:**
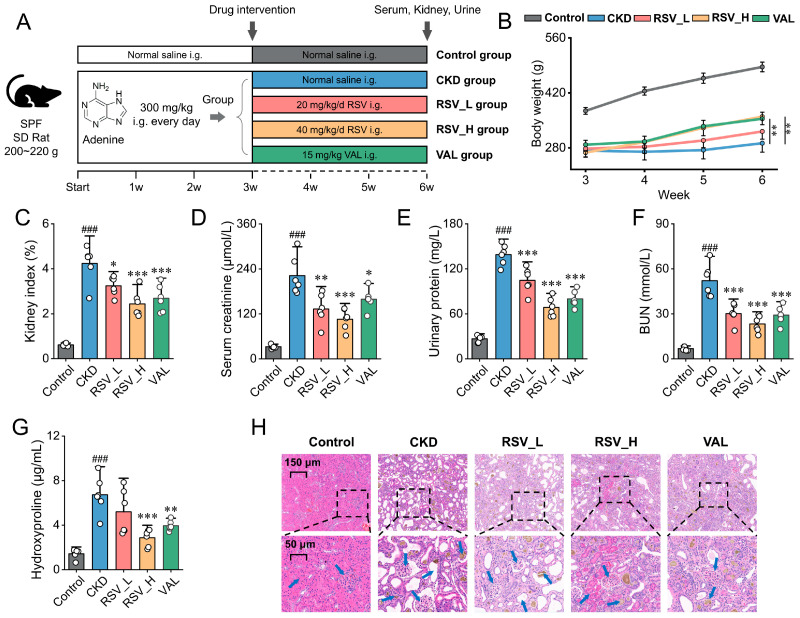
Resveratrol ameliorates adenine-induced CKD in rats. (**A**) Flowchart of animal experiments (**B**) Body weight changes during modeling and treatment. (**C**) Kidney index. (**D**–**F**) Renal function indicators: serum creatinine (**D**), urinary protein (**E**), and BUN (**F**). (**G**) Serum hydroxyproline level. (**H**) Representative H&E-stained kidney sections showing adenine-induced tubular injury/inflammation and crystal deposition, which were alleviated by resveratrol (scale bars: 150 μm and 50 μm). Data are presented as mean ± SD (*n* = 6/group). ^###^ *p* < 0.001 vs. Control; * *p* < 0.05, ** *p* < 0.01, *** *p* < 0.001 vs. CKD.

Given the well-documented toxicity of Triptolide, including its narrow therapeutic window and dose-limiting adverse effects, Resveratrol was selected for subsequent intervention studies because of its relatively favorable safety profile. The CKD rat model was established by intragastric administration of adenine for 21 days, followed by a 3-week Resveratrol intervention (Figure 6A). Model evaluation on Day 21 showed that, compared with the Control group, rats in the CKD group exhibited significant body weight loss (*p* < 0.001, Figure S4A). Meanwhile, serum creatinine and BUN levels were markedly increased (both *p* < 0.001, Figure S4B,C), and urinary protein levels were also significantly elevated (*p* < 0.001), indicating substantial renal dysfunction and confirming the successful establishment of the adenine-induced CKD model. Over the full treatment course, compared with the CKD group, both Resveratrol and Valsartan interventions significantly ameliorated the trend of body weight loss (Figure 6B) and reduced the kidney index (Figure 6C). Analysis of serum and urinary biochemical markers showed that both Resveratrol and Valsartan significantly reduced serum creatinine, BUN, and urinary protein levels (Figure 6D–F), as well as renal hydroxyproline content (Figure 6G), compared with the CKD group. Notably, RSV_H was similar to Valsartan on physical indicators and conventional renal function markers (serum creatinine, BUN, and urinary protein), as well as on the fibrosis marker hydroxyproline. Histopathological examination showed that the Control group maintained normal renal architecture, whereas the CKD group exhibited marked tubular dilation, structural disruption, interstitial expansion with inflammatory cell infiltration, glomerular atrophy, and brownish adenine crystal deposition. These pathological alterations were attenuated after Resveratrol and Valsartan treatment, with more pronounced improvements observed in the RSV_H and VAL group (Figure 6H).

### 2.7. Resveratrol Attenuates Renal Fibrosis and Downregulates c-Jun and C3 Protein Expression in Adenine-Induced CKD Rats

**Figure 7:**
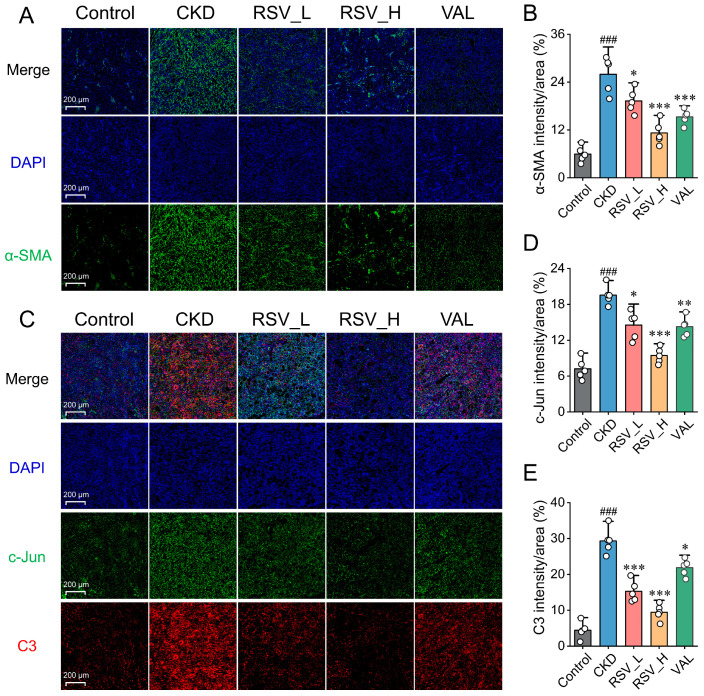
Resveratrol attenuates renal fibrosis and suppresses c-Jun and C3 protein expression in adenine-induced CKD rats. (**A**) Representative immunofluorescence images of α-SMA (green) with DAPI nuclear counterstaining (blue) in renal sections. (**B**) Quantification of α-SMA fluorescence intensity/area. (**C**) Representative immunofluorescence images of c-Jun (green) and C3 (red) with DAPI (blue). (**D**,**E**) Quantification of c-Jun and C3 fluorescence intensity/area. Data are presented as mean ± SD (*n* = 5/group). ^###^ *p* < 0.001 vs. Control; * *p* < 0.05, ** *p* < 0.01, *** *p* < 0.001 vs. CKD.

To further evaluate the renoprotective effects of Resveratrol in CKD rats, immunofluorescence staining was performed to assess the expression of the fibrosis marker α-SMA. Compared with the Control group, fluorescence intensity in the renal interstitium was markedly increased in the CKD group (Figure 7A,B). However, both Resveratrol and Valsartan interventions significantly reduced α-SMA expression, suggesting that both treatments attenuated adenine-induced renal fibrosis. Notably, RSV_H was similar to Valsartan in reducing α-SMA expression. We next examined the expression and distribution of c-Jun and C3 proteins, which are encoded by the key genes *JUN* and C3, in renal tissues. As shown in Figure 7C–E, c-Jun and C3 protein levels were significantly increased in the CKD group compared with the Control group (*p* < 0.05). After Resveratrol and Valsartan intervention, the elevated expression of c-Jun and C3 in the adenine-induced CKD rat model was significantly reduced (*p* < 0.01). Notably, RSV_H showed a more pronounced inhibitory effect on c-Jun and C3 expression than Valsartan.

### 2.8. Resveratrol Partially Reverses Transcriptomic Alterations in Adenine-Induced CKD Rats

**Figure 8:**
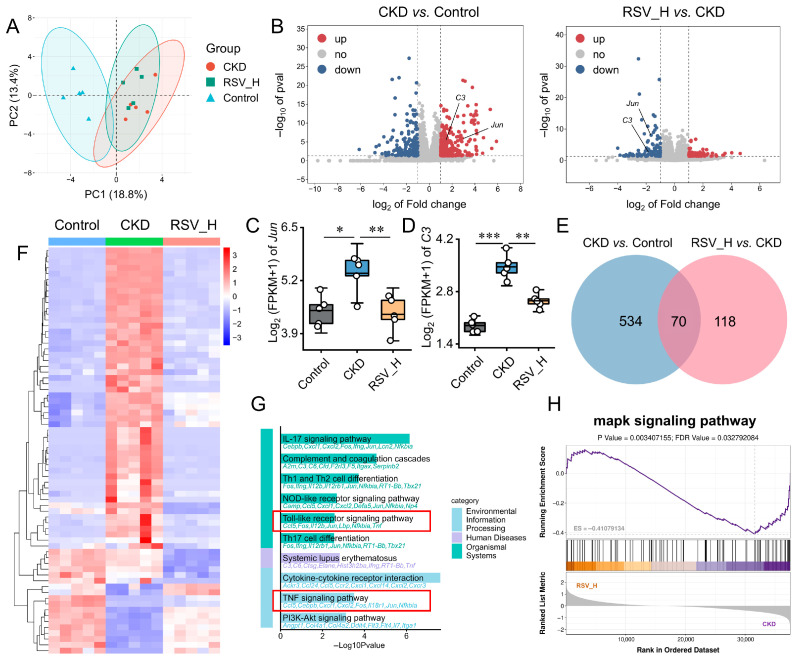
Resveratrol reverses CKD-associated transcriptomic signatures in rat kidney. (**A**) PCA showing distinct clustering among the Control, CKD, and RSV_H groups. (**B**) Volcano plot of DEGs in CKD vs. Control and RSV_H vs. CKD, with *Jun* and *C3* highlighted. (**C**,**D**) Boxplots showing RNA-seq expression levels of *Jun* (**C**) and *C3* (**D**) across groups. (**E**) Venn diagram showing the overlap of DEGs between “CKD vs. Control” and “RSV_H vs. CKD”. (**F**) Heatmap of overlapping genes with expression patterns reversed by resveratrol treatment. (**G**) KEGG enrichment analysis of DEGs in RSV_H vs. CKD, highlighting inflammation-related pathways (**H**). GSEA showing suppression of the MAPK signaling pathway in RSV_H vs. CKD. Orange indicates genes positively associated with the RSV_H group, whereas purple indicates genes positively associated with the CKD group. Data are presented as mean ± SD (*n* = 5/group). * *p* < 0.05, ** *p* < 0.01, *** *p* < 0.001.

To evaluate transcriptional changes in key genes, RNA-seq was performed on kidney tissues from the Control, CKD, and RSV_H groups. PCA showed distinct clustering and clear separation among the three groups (Figure 8A). Differential expression analysis showed that, compared with the Control group, the CKD group had 354 upregulated and 250 downregulated genes, with *Jun* and *C3* significantly upregulated (Figure 8B). In the RSV_H versus CKD comparison, 77 genes were upregulated and 111 genes were downregulated. Notably, Resveratrol intervention significantly reduced *Jun* and *C3* expression compared with the CKD group (Figure 8C,D). Intersection analysis of DEGs from the “CKD vs. Control” and “RSV_H vs. CKD” comparisons identified 70 overlapping genes (Figure 8E), among which 54 genes showed reversed expression patterns after Resveratrol treatment, as shown in the heatmap (Figure 8F).

To further explore the molecular mechanisms underlying the effects of Resveratrol in CKD, KEGG enrichment analysis was performed on treatment-related DEGs. The results showed significant enrichment of the TNF signaling pathway and NOD-like receptor signaling pathway (Figure 8G), both of which are closely associated with inflammatory activation and may regulate downstream MAPK-related signaling. Consistently, GSEA further revealed significant downregulation of the MAPK signaling pathway after Resveratrol intervention (Figure 8H). Together with the enrichment results from human CKD datasets, these findings suggest that MAPK/AP-1-related signaling may participate in CKD-associated inflammatory and fibrotic responses and may represent a potential pathway affected by Resveratrol treatment in the adenine-induced CKD rat model.

### 2.9. Resveratrol Suppresses MAPK/JNK–c-Jun Signaling Activation in Adenine-Induced CKD Rats

**Figure 9:**
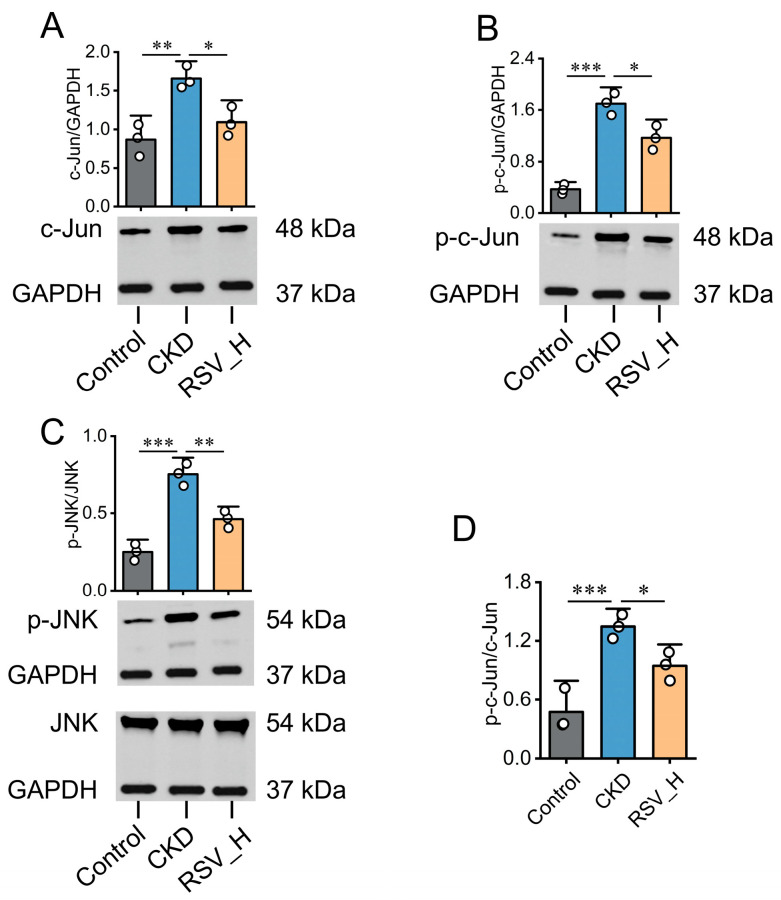
Resveratrol suppresses the JNK/c-Jun signaling pathway in adenine-induced CKD rats. (**A**,**B**) Representative Western blot bands and quantification of c-Jun (**A**) and p-c-Jun (**B**) protein expression in renal tissues. (**C**) Representative Western blot bands and quantification of the p-JNK/JNK ratio. (**D**) Quantification of the p-c-Jun/c-Jun ratio. GAPDH was used as the loading control. Data are presented as mean ± SD (*n* = 3/group). * *p* < 0.05, ** *p* < 0.01, *** *p* < 0.001.

Transcriptome analysis indicated that the MAPK signaling pathway may play an important role in CKD progression. Among its major branches, the JNK signaling pathway is highly responsive to cellular stress, inflammation, and apoptosis and can activate AP-1 transcriptional activity through c-Jun phosphorylation, thereby promoting the transcription of downstream inflammatory and fibrotic mediators. Based on these findings, we hypothesized that Resveratrol may exert its protective effects in CKD by modulating the MAPK/JNK signaling axis. Therefore, the expression levels of JNK, c-Jun, and their phosphorylated forms, p-JNK and p-c-Jun, were examined by Western blot. The results showed that total c-Jun protein expression (Figure 9A), p-c-Jun levels (Figure 9B), the p-JNK/JNK ratio (Figure 9C), and the p-c-Jun/c-Jun ratio (Figure 9D) were significantly increased in the CKD group. After Resveratrol intervention, these parameters were reduced to varying degrees, suggesting that Resveratrol may inhibit aberrant activation of JNK/c-Jun signaling axis, thereby contributing to the attenuation of renal fibrosis in CKD.

## 3. Discussion

The pathogenesis of CKD is highly complex, involving interconnected regulatory networks composed of multiple genes and signaling pathways. However, highly specific biomarkers for early diagnosis and safe, effective therapeutic agents remain limited [16]. In this study, we integrated bioinformatics analysis, machine learning screening, and reverse network pharmacology prediction to identify candidate biomarkers associated with CKD and explore potential therapeutic compounds. Through the combinatorial application of WGCNA and three machine learning algorithms, *JUN* and C3 were identified as potential diagnostic biomarker candidates for CKD. Reverse network pharmacology further identified Resveratrol as a candidate active compound potentially targeting these core genes. In vivo experiments showed that Resveratrol ameliorated adenine-induced renal fibrosis and renal dysfunction in rats, and this effect was associated with reduced activation of the MAPK/JNK signaling axis.

Using WGCNA, we constructed a weighted gene co-expression network. Within this network, the Turquoise module positively correlated with CKD, whereas the Red module showed a negative correlation. Both modules were primarily enriched in biological processes associated with immune-inflammatory activation and the suppression of mitochondrial metabolism. CIBERSORT immune infiltration analysis further supported this feature, showing increased infiltration of M1 macrophages, activated CD4^+^ memory T cells, and activated mast cells in CKD tissues, accompanied by a marked reduction in CD8^+^ T cells. M1 macrophages are known to secrete pro-inflammatory cytokines, such as IL-6 and TNF-α, thereby promoting renal inflammation and fibrosis progression [9]. This finding is consistent with the activation of chemokine- and cytokine receptor-related pathways identified by GSEA. Furthermore, the three machine learning algorithms identified *JUN* and C3 as candidate biomarkers associated with CKD, both of which showed promising diagnostic potential in the training dataset and external validation cohort. While established emerging biomarkers such as NGAL (encoded by LCN2) and KIM-1 (encoded by HAVCR1) are widely recognized in the context of kidney injury, we further explored their transcriptomic expression alongside *JUN* and C3 to contextualize our findings. Interestingly, within the specific datasets analyzed in this study, *JUN* (AUC: 0.976 and 0.952) and C3 (AUC: 0.809 and 0.828) exhibited relatively higher and more stable discriminatory capabilities compared to HAVCR1 (AUC: 0.778 and 0.761) and LCN2 (AUC: 0.639 and 0.655) across both the GSE175759 training and GSE37171 validation cohorts. These transcriptomic comparisons rely on retrospective tissue data and cannot fully replace clinical models that use continuous urinary or serological parameters. Nevertheless, *JUN* and C3 performed well within our analyzed datasets. This favorable performance underscores their diagnostic potential and supports their relevance as promising candidates for future prospective evaluation (Figure S5).

Previous studies have demonstrated that C3 is a central component of the complement system. Excessive activation of the alternative complement pathway can lead to the deposition of C3 cleavage products, such as C3a and C3b, in glomerular tissues, thereby inducing mesangial cell proliferation and inflammatory cascades. This mechanism is particularly prominent in complement-mediated kidney diseases, including C3 glomerulopathy and IgA nephropathy [17]. In recent clinical studies, C3 inhibitors, such as Pegcetacoplan, and Factor B inhibitors, such as Iptacopan, have been reported to reduce proteinuria and slow renal function decline in complement-mediated kidney diseases [17,18]. These findings support the pathogenic relevance of complement activation in kidney disease and further strengthen the biological plausibility of C3 as a CKD-associated candidate biomarker.

Beyond the complement system, c-Jun, a core component of the activator protein-1 (AP-1) transcription factor encoded by *JUN*, also plays an important role in CKD progression [19]. Multiple experimental studies have shown that aberrant activation of c-Jun-related signaling contributes to renal fibrosis and inflammatory responses [20]. In the unilateral ureteral obstruction (UUO) model, c-Jun phosphorylation is positively associated with the severity of tubulointerstitial fibrosis. Notably, the JNK inhibitor CC-401 significantly attenuates renal fibrosis by reducing myofibroblast accumulation and collagen IV deposition [21]. In diabetic nephropathy, c-Jun overexpression has been reported to promote mesangial cell proliferation through activation of the JNK pathway, whereas the c-Jun inhibitor SR11302 can ameliorate glomerulosclerosis [22,23]. In addition, epigenetic studies have suggested that HDAC inhibitors may suppress *JUN* expression by modulating promoter acetylation [24]. SGLT2 inhibitors have also been reported to exert cardiorenal protective effects, potentially involving c-Jun-related regulatory mechanisms, further supporting the relevance of c-Jun in the pathological network of CKD [21,25,26].

Despite the robust findings in our animal model, we observed a critical discrepancy regarding *JUN* expression between clinical transcriptomic databases and our in vivo data. In the adenine-induced CKD rat model, *Jun* mRNA and c-Jun protein levels (including phosphorylated c-Jun) were significantly upregulated. Conversely, our analysis of multiple human CKD datasets from the GEO database consistently revealed a significant downregulation of *JUN* mRNA. To address this apparent species divergence and the discrepancy between transcript and protein levels, it is helpful to distinguish between bulk mRNA abundance and functional protein activation. Transcription factors like c-Jun are frequently regulated via post-translational modifications (e.g., phosphorylation) and nuclear translocation, meaning baseline mRNA levels may not fully reflect their functional status. Importantly, existing clinical evidence suggests a pattern of c-Jun protein activation in human renal disease that contrasts with the transcriptomic downregulation trend. For instance, De Borst et al. observed the induction and nuclear localization of c-Jun protein in both the glomeruli and tubules of human renal disease biopsies [27]. Similarly, Mezzano et al. reported tubular activation of AP-1—of which c-Jun is a core component—in human proteinuric renal disease [28]. Furthermore, c-Jun has been implicated in acting synergistically with SP1 to promote TGFβ1-mediated disease progression in human diabetic nephropathy [29]. These clinical protein-level observations are broadly consistent with the upregulated c-Jun protein status observed in our rat model. Therefore, we hypothesize that the downregulation of *JUN* mRNA in retrospective GEO datasets (which predominantly feature ESRD samples) might reflect a severe loss of functional cells, tissue exhaustion, or a negative feedback response to chronic protein hyperactivation, rather than a definitive absence of c-Jun pathological signaling in humans.

In this context, the adenine-induced model employed in our study serves to simulate the active progression phase of CKD. This phase is characterized by intense inflammatory responses and high fibroblast activation [30], a state that likely involves the sustained activation of the AP-1/c-Jun pathway to drive the fibrotic process. This dynamic aligns with previous studies indicating that c-Jun expression undergoes a rapid upregulation during early renal injury and compensatory growth phases [31]. Following Resveratrol intervention, the overexpression and activation of c-Jun were significantly attenuated, acting in tandem with improvements in renal function and the amelioration of fibrosis. Supported by our in vivo findings and the clinical literature highlighting human c-Jun protein activation [27,28], these results suggest that the hyperactivation of the c-Jun axis may be an important pathological link contributing to CKD progression. Consequently, the ability of Resveratrol to attenuate this high expression implies that c-Jun represents a plausible mechanistic target for this intervention, though further prospective clinical studies are warranted to fully elucidate these cross-species regulatory differences.

In summary, the involvement of *JUN* and C3 in CKD progression supports their potential relevance as promising diagnostic candidates and therapeutic targets, but does not establish their clinical utility as validated early diagnostic biomarkers. In contrast to the traditional “drug-seeking-target” paradigm of network pharmacology, this study adopted a more focused “target-seeking-drug” reverse strategy to identify active compounds potentially targeting the core genes implicated in CKD-related pathological mechanisms. Using this strategy, we identified two natural bioactive compounds that may concurrently target *JUN* and C3: Triptolide and Resveratrol. Triptolide, the major active monomer of *Tripterygium wilfordii*, has been reported to exert potent anti-inflammatory and immunosuppressive effects and to delay disease progression in experimental models of polycystic kidney disease and glomerulonephritis. However, its clinical translation is limited by its narrow therapeutic window and potential hepatic, renal, and reproductive toxicities [32,33]. In contrast, Resveratrol, a natural polyphenol widely found in plants such as *Polygonum cuspidatum*, was selected as the preferred candidate for further investigation because of its more favorable safety profile and pleiotropic biological activities.

Existing studies have demonstrated the substantial potential of Resveratrol in CKD. In vivo evidence indicates that Resveratrol exerts broad renoprotective effects across several classical animal models of kidney injury. In a 5/6 nephrectomy model, Resveratrol attenuates oxidative stress by activating Sirt1 and enhancing Sirt1–FoxO1 interaction, thereby upregulating MnSOD expression. It also inhibits the TGF-β/Smad2/3 signaling pathway, reducing collagen deposition and tubulointerstitial fibrosis and improving left ventricular remodeling [34]. In diabetic nephropathy models, Resveratrol protects podocytes and reduces proteinuria by upregulating nephrin and WT1 expression [35]. It also decreases renal inflammatory mediators, such as ICAM-1, by suppressing SphK1 expression and activating autophagy-related pathways [36]. In addition, Resveratrol has been shown to improve lipid metabolism, reduce serum uric acid levels, and alleviate basement membrane thickening in models of membranous nephropathy and hyperuricemic nephropathy, supporting its multi-target and multi-pathway renoprotective properties [37]. Mechanistically, Resveratrol acts as a natural Sirt1 agonist and exerts protective effects through multiple pathways, including activation of the Sirt1–FoxO1–MnSOD antioxidant axis, modulation of the Sirt1–Smad3 anti-fibrotic pathway, and inhibition of NF-κB/NLRP3 inflammasome activation [34]. These mechanisms are closely related to *JUN*/c-Jun-mediated transcriptional regulation, which was identified as a core molecular feature in our study. Previous studies have shown that Sirt1 can directly deacetylate AP-1 and suppress its transcriptional activity [38], providing a plausible mechanistic link between Resveratrol intervention and the regulation of *JUN*-related signaling. Our in vivo experimental results provide supporting evidence for this logical link; Resveratrol intervention attenuated the aberrant activation of the MAPK/JNK pathway in the kidney tissues of CKD rats and downregulated c-Jun phosphorylation levels, which was accompanied by the mitigation of renal fibrosis. These findings are not only consistent with our reverse network pharmacology predictions but also further suggest that Resveratrol may exert its protective effects in the core pathological network of CKD partly by modulating the MAPK/JNK signaling axis. Furthermore, when compared to Valsartan, high-dose Resveratrol was similar on renal function and macroscopic fibrosis markers (α-SMA, hydroxyproline), but stronger on suppressing targeted c-Jun and C3 protein expression. From a translational perspective, the rat doses of Resveratrol used in this study correspond approximately to human equivalent doses of 190–390 mg/day for a 60 kg adult based on body surface area conversion; however, its low oral bioavailability remains a major limitation, and further pharmacokinetic and clinical validation is still required.

While this study offers novel strategies for the diagnosis and treatment of CKD, several limitations must be acknowledged. Our human data relies on retrospective biopsies from established CKD or ESRD, reflecting late-stage tissue remodeling. Conversely, our in vivo validation uses a short-term, rapidly progressing adenine-induced rat model. Because neither model captures the chronic, subclinical onset of early human CKD, *JUN* and C3 should be strictly interpreted as CKD-associated candidate biomarkers rather than definitive early diagnostic markers. Second, the small control sample in GSE66494 limited reliable ROC-based evaluation, so this dataset was used only for supplementary expression validation. Third, relying on a single, male-only animal model and invasive tissue-level samples cannot fully represent the broad etiological and sex-based heterogeneity of human CKD. Therefore, further validation in diverse prospective clinical cohorts using accessible biofluids (e.g., serum or urine) is essential.

## 4. Materials and Methods

### 4.1. Materials and Reagents

Adenine (HY-B0152), sodium carboxymethyl cellulose (HY-Y0703), resveratrol (HY-16561), and valsartan (HY-18204) were purchased from MedChemExpress (Monmouth Junction, NJ, USA). The serum creatinine assay kit (0065-2007) was purchased from Yonghe Sunshine Biotechnology Co., Ltd. (Changsha, China), the blood urea nitrogen assay kit (AF002742) from Aifang Biotechnology Co., Ltd. (Changsha, China), the hydroxyproline assay kit (YX-W-A907) from Youxuan Biotechnology Co., Ltd. (Shanghai, China), and the urine protein assay kit (E-BC-K252-M) from Elabscience (Wuhan, China); these assays were performed utilizing a fully automated biochemical analyzer (7180, Hitachi, Tokyo, Japan) and a microplate reader (IF200, Tecan, Switzerland). Regarding antibodies, anti-α-SMA (1:1000, 20250918), anti-JNK (CYRM30488), anti-phosphorylated JNK (p-JNK, AF00815XX), anti-c-Jun (1:1000, CSGL250526), anti-phosphorylated c-Jun (p-c-Jun, CYRM0436), and anti-C3 (1:1000, CSXL251015) were all purchased from Aifang Biotechnology Co., Ltd. (Changsha, China). All other routine reagents not specifically indicated were of analytical or biochemical grade.

### 4.2. Data Processing

Using “Chronic Kidney Disease” and “CKD” as search terms, we downloaded CKD-related gene expression datasets (GSE175759, GSE37171, and GSE66494) from the Gene Expression Omnibus (GEO; https://www.ncbi.nlm.nih.gov/geo/; search cut-off date: 20 October 2025). The GSE175759 dataset comprised renal biopsy specimens from 43 cases of IgA nephropathy, 3 cases of diabetic nephropathy, 3 cases of focal segmental glomerulosclerosis, 3 cases of lupus nephritis, 4 cases of membranous nephropathy, and 9 cases of minimal change disease, along with 22 healthy control samples. The GSE37171 dataset included samples from 63 patients with end-stage renal disease and 20 healthy controls, whereas GSE66494 contained renal biopsy samples from 53 patients with chronic kidney disease and 5 healthy controls. GSE175759 was used as the internal training set, and GSE37171 and GSE66494 were used as external validation sets. Raw CEL files were processed using the R package “affy”, and the robust multi-array average (RMA) algorithm was applied for background correction, quantile normalization, and probe-level summarization. Probe identifiers were mapped to official gene symbols, and the mean expression across multiple probes targeting the same gene was calculated. To ensure data quality, low-expression genes were filtered out, retaining only genes with an FPKM > 1 in at least 50% of the samples. The data was subsequently Log2 transformed.

### 4.3. Identification of Differentially Expressed Genes (DEGs)

Differential expression analysis was performed using the “limma” package in R to identify DEGs between CKD patients and healthy controls. DEGs were defined using the following criteria: fold change (FC) > 1.5 and false discovery rate (FDR)-adjusted *p*-value < 0.05.

### 4.4. Weighted Gene Co-Expression Network Analysis

Weighted gene co-expression networks were constructed using the “WGCNA” package in R to identify key gene modules associated with CKD. First, sample and gene quality were evaluated using the goodSamplesGenes function, and outlier samples or genes were removed before network construction. An appropriate soft-thresholding power (β) was selected by evaluating network topology; β = 6 was finally chosen because it satisfied the criteria for a scale-free distribution. The blockwise network construction and module detection were performed using the blockwiseModules function with an unsigned topological overlap matrix (TOM) (TOMType = “unsigned”). Genes were hierarchically clustered using average linkage. To assign genes with similar expression patterns to distinct co-expression modules, the main parameters were set as follows: minModuleSize = 50, mergeCutHeight = 0.25, and deepSplit = 2. To identify the modules most relevant to the CKD phenotype, Pearson correlations and corresponding Student’s *p*-values were calculated between module eigengenes (MEs) and clinical trait data. Modules exhibiting the highest absolute correlation coefficients and statistical significance (*p* < 0.05) were selected as key CKD-associated modules for subsequent downstream analyses, and module–trait relationships were visualized using a heatmap.

### 4.5. Identification of Intersecting Genes and Functional Enrichment Analyses

DEGs identified in Section 2.3 were intersected with genes from the module showing the strongest association in Section 2.4, and the overlap was visualized using a Venn diagram. Using the “clusterProfiler” package in R together with the human gene annotation database org.Hs.eg.db, we performed systematic functional annotation and enrichment analyses of the intersecting genes, including Gene Ontology (GO) annotation, Kyoto Encyclopedia of Genes and Genomes (KEGG) pathway analysis, and gene set enrichment analysis (GSEA). For all enrichment analyses, an adjusted *p*-value < 0.05 was considered statistically significant.

### 4.6. Construction of the PPI Network

To further identify key regulatory targets among the intersecting genes, PPI analysis was performed for proteins encoded by these genes using the STRING database (https://cn.string-db.org/; accessed on 25 October 2025). The organism was set to “Homo sapiens”, and a minimum interaction confidence score > 0.7 was applied to retain high-confidence interactions. The resulting PPI data were exported in TSV format and imported into Cytoscape (v3.10.1) for visualization and topological analysis. Isolated nodes without edges were removed, and only protein nodes with meaningful interactions were retained. The Cytoscape plug-in CentiScaPe 2.2 was then used to identify hub targets by applying thresholds based on topological parameters, including degree, closeness centrality, and betweenness centrality.

### 4.7. Immune Cell Infiltration Analysis

To systematically evaluate changes in the immune microenvironment in CKD, we applied a gene expression-based deconvolution approach using the CIBERSORT R package to quantify immune cell infiltration, characterize differences in immune cell composition and infiltration levels between CKD patients and healthy controls, and visualize the distribution of immune cell fractions in the two groups using boxplots. Differences in immune cell proportions between groups were further assessed using the Wilcoxon rank-sum test, with *p* < 0.05 considered statistically significant.

### 4.8. Machine Learning Analysis

Machine-learning analysis was performed to identify promising diagnostic candidates for CKD. The feature space used for model construction consisted of the 26 hub genes identified from the PPI network. The GSE175759 dataset was used as the training cohort. Detailed parameter settings and implementation procedures for LASSO, SVM-RFE, and Random Forest analyses are provided in the Supplementary Materials.

### 4.9. Nomogram Construction and Receiver Operating Characteristic (ROC) Curve Evaluation

To evaluate the predictive capability of the identified hub genes for CKD, a diagnostic nomogram was constructed using the rms package in R4.5.1 based on the finalized gene signature. The nomogram assigned scores to each gene according to its regression coefficient and converted the total score into a predicted probability of CKD. ROC curves were then generated to assess discriminatory performance. For model-level evaluation, ROC curves were generated using the prediction scores of the LASSO, Random Forest, and SVM classifiers in the external validation cohort GSE37171. For single-gene evaluation, the normalized expression values of *JUN* and C3 were used directly as predictors to generate ROC curves in GSE175759 and GSE37171. The Area Under the Curve (AUC) was calculated to quantify discriminatory capability, with an AUC value > 0.7 considered to indicate acceptable discriminatory ability. Estimated 95% confidence intervals (CIs) were added to the ROC plots for visual reference.

### 4.10. Reverse Screening of Active Compounds for Key Targets

To investigate potential active compounds capable of modulating the identified key targets, a reverse network pharmacology approach was employed in this study. Known compounds interacting with these targets were retrieved through a systematic search of public databases, including TCMSP, HERB, BATMAN-TCM, and DrugBank. To ensure data reliability, only compounds supported by experimental validation or high predictive confidence scores were retained. Subsequently, candidate compounds were screened based on criteria of Oral Bioavailability (OB) ≥ 30% or Drug-Likeness (DL) ≥ 0.18 to guarantee their potential druggability. Finally, a “compound-target” interaction network was constructed and visualized using Cytoscape software (version 3.10.1).

### 4.11. Molecular Docking

The 3D structures of candidate small molecule ligands were retrieved from the PubChem database (http://pubchem.ncbi.nlm.nih.gov/) and saved in MOL2 format. Crystal structures of the target proteins were obtained from the Protein Data Bank (PDB, http://www.rcsb.org/); structures exhibiting high resolution, high human homology, and complete binding domains were selected as receptors. Receptor structures were pre-processed using PyMOL3.1.0 software. This process included removing crystal water molecules, non-essential ligands, and irrelevant phosphate groups, followed by adding hydrogen atoms and optimizing charges. Subsequently, molecular docking simulations were performed using AutoDock Vina (version 1.5.6), and the protein-ligand complexes exhibiting the optimal predicted binding modes were visualized using Discovery Studio 2019.

### 4.12. Molecular Dynamics Simulation

Molecular dynamics (MD) simulations were conducted using GROMACS 2022 software for a total duration of 100 ns, with detailed methodological procedures provided in the Supplementary Materials.

### 4.13. Establishment of the Adenine-Induced CKD Model in SD Rats

Thirty-six healthy male SD rats (8–12 weeks old, 200–220 g) were purchased from Hunan SJA Laboratory Animal Co., Ltd. (Changsha, China; License No. SCXK [湘] 2021-0002). All rats were housed in a barrier environment at the Animal Experiment Center of the Hunan Academy of Chinese Medicine, with ad libitum access to food and water. Following acclimatization, the rats were randomly divided into two groups: the normal control group (Control group, *n* = 9) and the chronic kidney disease model group (CKD, *n* = 27). The CKD model was induced via intragastric administration of adenine. First, adenine powder was suspended in a 1.5% sodium carboxymethyl cellulose (CMC-Na) solution to achieve a concentration of 30 mg/mL. Rats in the model group were then administered this suspension via oral gavage (10 mL/kg, equivalent to 300 mg/kg/day of adenine) once daily for 21 consecutive days. The Control group received an equivalent volume of the 1.5% CMC-Na solution.

Upon completion of the modeling period (Day 21), three rats were randomly selected from both the Control and model groups for preliminary model evaluation. The rats were placed in metabolic cages to collect 24 h urine samples. Subsequently, the rats were anesthetized via intraperitoneal injection of pentobarbital sodium (30 mg/kg), and whole blood was collected from the abdominal aorta prior to euthanasia. Bilateral kidneys were rapidly harvested and weighed to calculate the kidney index (kidney weight/body weight × 100). The remaining 24 successfully modeled rats were randomly allocated into four groups (*n* = 6 per group): the CKD group, the low-dose Resveratrol group (RSV_L, 20 mg/kg), the high-dose Resveratrol group (RSV_H, 40 mg/kg), and the positive control group (Valsartan, VAL, 15 mg/kg). During this phase, the CKD and Control groups continued to receive equal volumes of CMC-Na solution, while the treatment groups were administered the corresponding drug dosages via oral gavage once daily for 3 weeks. Following the final administration, 24 h urine samples were collected from all rats. Rats were then anesthetized, blood samples were collected, and euthanasia was performed as described previously to harvest kidney tissues. One kidney was perfused with pre-cooled PBS and fixed in 4% paraformaldehyde for subsequent histological and immunofluorescence analyses, while the contralateral kidney was snap-frozen in liquid nitrogen and stored at −80 °C. All animal experimental protocols in this study were reviewed and approved by the Animal Ethics Committee of the Hunan Academy of Chinese Medicine (Ethics No. SY-2025-0130).

### 4.14. Determination of Serum Biochemical Indicators and Urinary Protein

Upon completion of the experiment, whole blood samples were collected from rats in each group and centrifuged to isolate serum; additionally, 24 h urine samples were obtained using metabolic cages. Renal function-related parameters, including serum creatinine, blood urea nitrogen (BUN), serum hydroxyproline, and urinary protein levels, were measured using an automatic biochemical analyzer. All assays were performed in strict accordance with the manufacturers’ instructions provided with the respective kits.

### 4.15. Histopathological Observation of Renal Tissue

Following the experiment, the right kidneys were rapidly excised, fixed in 4% paraformaldehyde, embedded in paraffin, and sectioned. Sections were stained with hematoxylin and eosin (H&E), and morphological changes in the renal tissue were examined using an optical microscope. The evaluation focused specifically on adenine-induced pathological features, including necrosis and shedding of tubular epithelial cells, tubular dilation, interstitial inflammatory cell infiltration, and the deposition of adenine crystals. Pathological images were acquired and analyzed using KFSlideOS1.0.8 software.

### 4.16. Immunofluorescence Staining

Paraffin-embedded sections first underwent deparaffinization, rehydration, and heat-induced antigen retrieval (EDTA, pH 9.0). The sections were then treated with 3% H_2_O_2_ to block endogenous peroxidase activity, and subsequently incubated with 10% goat serum to minimize non-specific binding. The sections were then incubated overnight at 4 °C with specific primary antibodies. On the following day, horseradish peroxidase (HRP)-conjugated secondary antibodies were applied, followed by signal detection using Tyramide Signal Amplification (TSA) technology (TYR-570/TYR-520). Nuclei were counterstained with DAPI, and slides were mounted with an antifade medium; pathological morphology of the renal tissue was visualized using a fluorescence microscope, and image analysis was performed using KFSlideOS software.

### 4.17. Transcriptome Sequencing Analysis

Total RNA was extracted from the kidney tissues of rats in each group using TRIzol reagent (Invitrogen, Carlsbad, CA, USA), and its concentration and quality were assessed using a NanoDrop ND-1000 spectrophotometer (Thermo Fisher Scientific, Wilmington, DE, USA). Detailed protocols for sample quality control, data filtering, and the bioinformatics analysis workflow are provided in the Supplementary Materials. DEGs were identified based on screening thresholds of *p* < 0.05 and FC > 2 or FC < 0.5.

### 4.18. Western Blot Analysis

Rat kidney tissues were homogenized in RIPA lysis buffer containing 1 mM PMSF to extract total protein. Protein concentration was accurately determined using the BCA assay, followed by thermal denaturation. Equal amounts of protein were separated via SDS-PAGE and subsequently transferred onto 0.45 µm PVDF membranes at a constant current of 350 mA. The membranes were blocked with 5% non-fat milk for 30 min and then incubated with primary antibodies at 4 °C overnight. The following day, membranes were washed with TBST and incubated with HRP-conjugated secondary antibodies (1:5000) for 30 min at room temperature. Finally, protein bands were visualized using an ECL chemiluminescence reagent, and quantitative analysis of the raw TIFF images was performed using AIWBwell^TM^ software (version 2.10).

### 4.19. Statistical Analysis and Image Processing

Data analysis was performed using SPSS 23.0 software. Quantitative data are expressed as mean ± standard deviation. Comparisons between two groups were assessed using the independent samples t-test, while differences among multiple groups were evaluated using one-way analysis of variance (ANOVA) followed by Tukey’s post hoc test. Furthermore, ImageJ 1.54r software was utilized to quantify gray values or positive areas in Western blot bands and immunofluorescence images. A *p*-value < 0.05 was considered statistically significant.

## 5. Conclusions

This study established an integrated screening strategy combining multi-omics mining with machine learning, identifying *JUN* and C3 as promising diagnostic candidates for CKD while unveiling the characteristic immune microenvironment dysregulation associated with the disease. A reverse network pharmacology approach suggested Resveratrol as a key active compound targeting these core genes. Computational simulations subsequently supported the stable binding affinity between Resveratrol and the target proteins. In vivo experiments demonstrated that Resveratrol significantly attenuated adenine-induced renal fibrosis, effectively improved renal function, and reversed pathological transcriptomic signatures. Mechanistically, we provide evidence that Resveratrol exerts its therapeutic effects in association with the downregulation of the MAPK/JNK signaling axis, thereby suppressing JNK-mediated c-Jun phosphorylation and its downstream cascades. Collectively, this study proposes a potential diagnostic and therapeutic strategy targeting *JUN*/C3, and suggests that Resveratrol may represent a promising preclinical intervention candidate for CKD, providing preliminary computational and preclinical evidence for CKD biomarker discovery and natural medicine-based intervention.

## Data Availability

Data will be made available on request.

## Supplementary Materials

The following supporting information can be downloaded at https://www.mdpi.com/article/10.3390/ijms27104252/s1.

## Abbreviations

The following abbreviations are used in this manuscript:

CKD	Chronic kidney disease
WGCNA	Weighted gene co-expression network analysis
PPI	Protein–protein interaction
DEGs	Differentially expressed genes
LASSO	Least absolute shrinkage and selection operator
SVM-RFE	Support vector machine-recursive feature elimination
GEO	Gene Expression Omnibus
GO	Gene Ontology
KEGG	Kyoto Encyclopedia of Genes and Genomes
GSEA	Gene set enrichment analysis
FC	Fold change
FDR	False discovery rate
RMSD	Root mean square deviation
RMSF	Root mean square fluctuation
Rg	Radius of gyration
SASA	Solvent accessible surface area
OB	Oral bioavailability
DL	Drug-likeness
Scr	Serum creatinine
BUN	Blood urea nitrogen
H&E	Hematoxylin and eosin
RSV	Resveratrol
AUC	Area under the curve
ROC	Receiver operating characteristic
TCM	Traditional Chinese medicine
NF-κB	Nuclear factor kappa B
TNF-α	Tumor necrosis factor-alpha
IL-6	Interleukin-6
IL-1β	Interleukin-1 beta
