# Supplementary material for: An Integrated Machine-Learning and Reverse Network-Pharmacology Pipeline Reveals JUN/C3 Candidate Biomarkers and an Anti-Fibrotic Mechanism of Resveratrol via MAPK/JNK Signaling in Chronic Kidney Disease
Source: Int J Mol Sci. 2026 May 10;27(10):4252. doi: 10.3390/ijms27104252 (PMC13206790; doi:10.3390/ijms27104252)
Supplement: Supplementary file 1 [file ijms-27-04252-s001.zip › ijms-4244697-supplementary.pdf]

Supporting information

An Integrated Machine-Learning and Reverse Network-Pharmacology Pipeline Reveals *JUN*/C3 Candidate Biomarkers and an Anti-Fibrotic Mechanism of Resveratrol via MAPK/JNK Signaling in Chronic Kidney Disease

Yuan Cai [1], Xiaolong Feng [1], Xinru Tao [1], Penghui Li [1], Jiaqin Liu [1], Ping’an Liu [1], Mengxiong Xiao [2,*]

[1] Hunan Academy of Chinese Medicine, Changsha, 410013, China

[2] Experimental Research Center, China Academy of Chinese Medical Sciences, Beijing, 100700, China

Materials and methods

1. Machine-learning parameter settings

For LASSO regression, the glmnet package was used to fit a binomial model, with alpha = 1 indicating L1 regularization. The optimal penalty parameter was selected using cv.glmnet with 5-fold cross-validation, and genes with non-zero coefficients at lambda.min were retained as candidate features. For Random Forest analysis, the randomForest package was used. The initial model was constructed with ntree = 500 and importance = TRUE. The number of trees corresponding to the minimum out-of-bag error was then selected, and the final Random Forest model was reconstructed using this optimized tree number. Gene importance was ranked according to the MeanDecreaseGini index, and the top-ranked genes were selected for subsequent analysis. For SVM-RFE analysis, the msvmRFE workflow was applied. A 10-fold cross-validation strategy was used to evaluate feature subsets, and the optimal number of features was determined according to the minimum classification error. Feature selection was performed across the 26 input genes, and the optimal feature subset was retained for downstream intersection analysis. No additional oversampling or undersampling strategy was applied for class imbalance. The final candidate diagnostic biomarkers were defined as genes consistently identified by LASSO, SVM-RFE, and Random Forest analyses.

2. Molecular dynamics simulation

In this study, molecular dynamics (MD) simulations were performed using GROMACS 2022. Force field parameters were obtained using the GROMACS pdb2gmx tool and the AutoFF web server. The AMBER14SB force field was applied to the receptor protein, while the GAFF2 force field was used for the ligand. The system was solvated in a cubic box of TIP3P water molecules. For large proteins such as C3, the box dimensions were strictly defined by maintaining a minimum distance of 1.0 nm between the protein surface and the periodic boundaries. This parameter choice ensures adequate solvation while effectively preventing artificial self-interactions across periodic images for such macromolecular complexes. Ions were then added using the gmx genion tool to neutralize the system. Long-range electrostatic interactions were treated using the Particle Mesh Ewald (PME) method with a cutoff radius of 1 nm. All bond constraints were applied using the SHAKE algorithm. An integration time step of 1 fs was employed, and the dynamics were propagated using the Verlet leapfrog algorithm. Prior to production MD simulations, the system was subjected to energy minimization. This process involved 3000 steps of steepest descent followed by 2000 steps of conjugate gradient minimization, carried out in three stages: (1) with the solute fixed and water molecules relaxed; (2) with counterions fixed; and (3) with no positional restraints applied to the entire system. The production simulation was conducted under an NPT ensemble at 310 K for a total duration of 100 ns. While a 100 ns simulation time is generally sufficient to evaluate the dynamic stability and local residue interactions of small-molecule ligands within their respective binding pockets, it is acknowledged as a limitation that this duration may not fully capture slow, large-scale allosteric conformational transitions across the entirety of a large protein structure like C3. Trajectory analysis was performed using GROMACS tools including gmx rmsd, gmx rmsf, gmx hbond, gmx gyrate, and gmx sasa to evaluate the root mean square deviation (RMSD), root mean square fluctuation (RMSF), number of hydrogen bonds, radius of gyration (Rg), and solvent accessible surface area (SASA), respectively.

3. Data filtering and analysis of transcriptomics analysis

Total RNA was isolated using TRIzol reagent (Thermo Fisher Scientific, 15596018). RNA integrity was assessed with a Qubit 3.0 Fluorometer (Thermo Fisher Scientific, Q33216) and Agilent 5300 Fragment Analyzer (Agilent Technologies, M5311AA), and only samples with RNA integrity number (RIN) > 7.0 were selected for downstream analysis. For library preparation, mRNA was purified from 2 μg total RNA through two rounds of selection using mRNA Capture Beads 2.0 (Yeasen Biotech, 12629ES). Purified mRNA was fragmented in magnesium-based buffer (Yeasen Biotech, 12340ES97) at 94°C, followed by first-strand cDNA synthesis with reverse transcriptase and second-strand synthesis using E. coli DNA polymerase I, RNase H, and dUTP solution. The fragmented cDNA underwent end repair, A-tailing, and ligation of Illumina dual-index adapters. PCR amplification was performed for 14 cycles (98°C for 1 min initial denaturation; 98°C for 10 sec, 60°C for 30 sec, 72°C for 30 sec per cycle; final extension at 72°C for 5 min) using high-fidelity DNA polymerase. Libraries with insert sizes of 400 ± 50 bp were purified with Hieff NGS DNA Selection Beads (Yeasen Biotech, 12601ES75) and sequenced on an Illumina NovaSeq X Plus platform (LC-Bio Technology) with 2 × 150 bp paired-end (PE150) configuration. Raw sequencing reads were filtered using Cutadapt (v1.11) to remove adapter-containing sequences, polyA/G tracts, reads with >5% ambiguous bases (N), and low-quality reads (Q-score ≤ 20). Data quality was verified with FastQC (v0.11.9) to assess Q20/Q30 scores and GC content. Clean data were deposited in the NCBI GEO/SRA databases under accession numbers GEO: < GEO accession > and SRA: < SRA accession >. Clean reads were aligned to the reference genome of the target species using HISAT2 (v2.2.1) with parameters allowing ≤ 2 mismatches and ≤ 20 multi-mapping positions per read. Splice junction databases were incorporated to enhance alignment accuracy. Transcript quantification was performed with StringTie (v2.1.6) and Ballgown to calculate FPKM (fragments per kilobase of transcript per million mapped reads) values. A comprehensive transcriptome was reconstructed by merging all sample-specific assemblies.

Figure Caption

Fig. S1. Correlation between module membership and gene significance in key WGCNA modules. (A) Turquoise module. (B) Red module. Each dot represents one gene; the correlation coefficient and *p*-value are shown in the plots.

Fig. S2. Nomogram model for predicting CKD risk based on JUN and C3.

Fig. S3. Single gene GSEA of JUN and C3 in CKD. (A) GSEA based on JUN expression. (B) GSEA based on C3 expression.

Fig. S4. Validation of the adenine-induced CKD rat model. (A) Body weight changes over 3 weeks. (B) Serum creatinine. (C) Urinary protein. (D) BUN. $^{***}p < 0.001$ *vs.* Control.

Fig. S5. Diagnostic performance of *LCN2* (encoding NGAL) and *HAVCR1* (encoding KIM-1) in the training and validation cohorts.

Fig. S1.

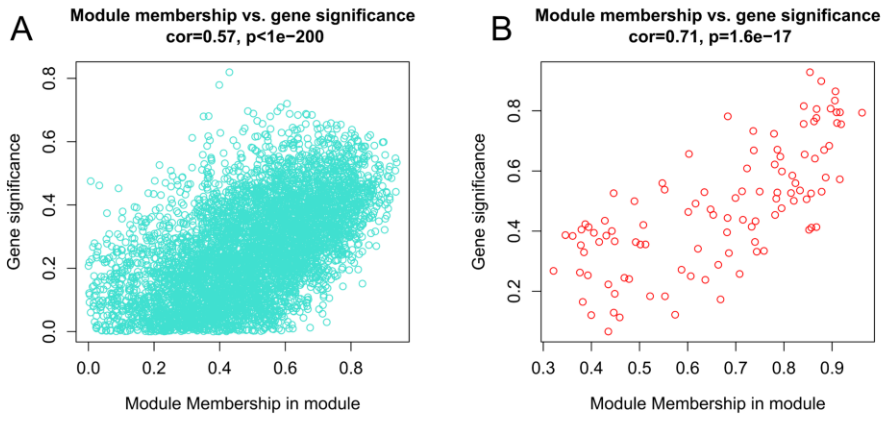

Fig. S2.

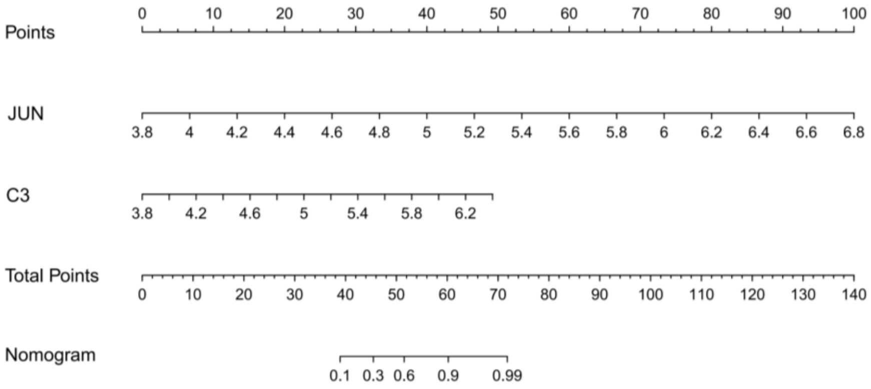

Fig. S3.

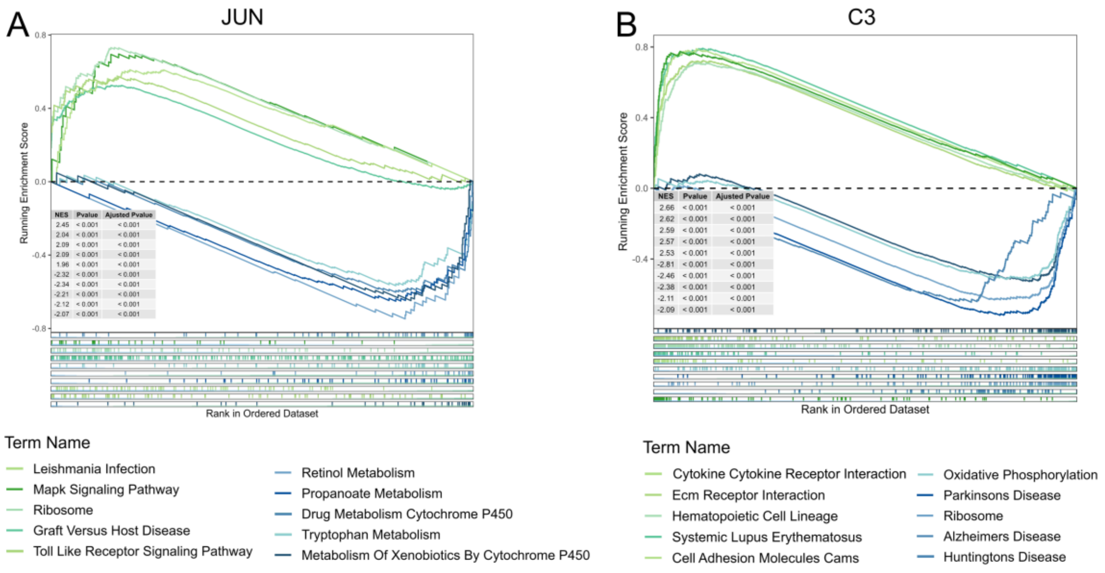

Fig. S4.

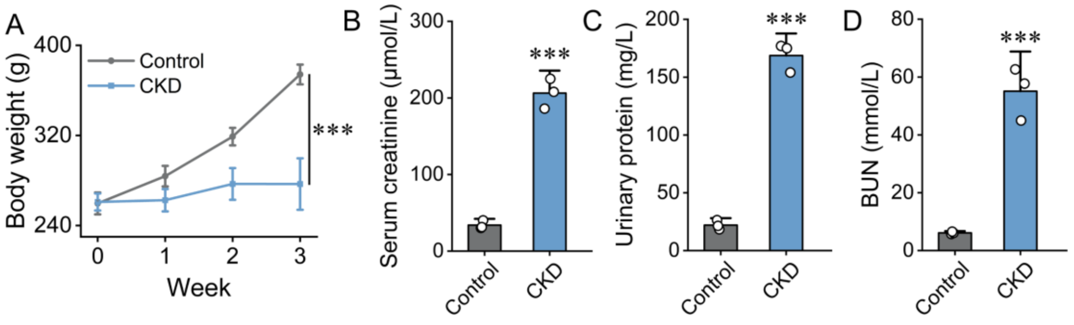
A
Body weight (g)
400
320
240
Control
CKD
***
0
1
2
3
Week
B
Serum creatinine (μmol/L)
300
200
100
0
***
Control
CKD
C
Urinary protein (mg/L)
200
150
100
50
0
***
Control
CKD
D
BUN (mmol/L)
80
60
40
20
0
***
Control
CKD

Fig. S5.

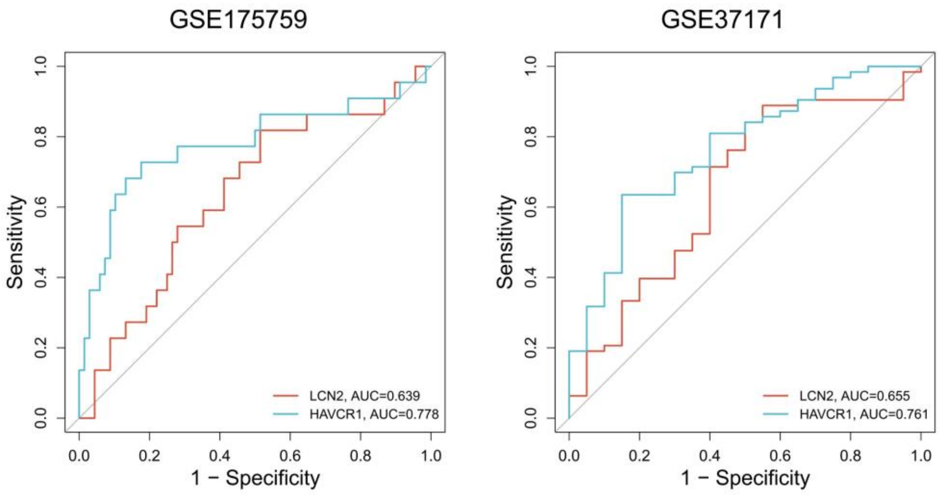
GSE175759
GSE37171
Sensitivity
1 − Specificity
LCN2, AUC=0.639
HAVCR1, AUC=0.778
LCN2, AUC=0.655
HAVCR1, AUC=0.761
0.0
0.2
0.4
0.6
0.8
1.0

Table Caption

Table. S1. Summary of ROC-based diagnostic performance for machine-learning models and candidate biomarkers.

Table. S1. Summary of ROC-based diagnostic performance for machine-learning models and candidate biomarkers.

Dataset	Model/Biomarker	AUC	SE	95% CL	*P* value	Sensitivity	Specificity	Youden index
GSE37171	LASSO	0.919	0.030	0.861–0.977	<0.001	1.00	0.80	0.80
GSE37171	Random Forest	0.851	0.042	0.768–0.934	<0.001	0.70	0.95	0.65
GSE37171	SVM	0.719	0.060	0.601–0.837	<0.001	0.72	0.70	0.42
GSE175759	JUN	0.976	0.015	0.948–1.000	<0.001	1.00	0.84	0.84
GSE175759	C3	0.809	0.047	0.716–0.902	<0.001	0.82	0.77	0.59
GSE37171	JUN	0.952	0.022	0.909–0.995	<0.001	0.94	0.85	0.79
GSE37171	C3	0.828	0.046	0.738–0.918	<0.001	0.85	0.70	0.55
